# Assessing Digital Phenotyping for App Recommendations and Sustained Engagement: Cohort Study

**DOI:** 10.2196/62725

**Published:** 2024-11-19

**Authors:** Bridget Dwyer, Matthew Flathers, James Burns, Jane Mikkelson, Elana Perlmutter, Kelly Chen, Nanik Ram, John Torous

**Affiliations:** 1 Division of Digital Psychiatry Beth Israel Deaconess Medical Center Harvard Medical School Boston, MA United States

**Keywords:** engagement, mental health, digital phenotype, pilot study, phenotyping, smartphone sensors, anxiety, sleep, fitness, depression, qualitative, app recommendation, app use, mobile phone

## Abstract

**Background:**

Low engagement with mental health apps continues to limit their impact. New approaches to help match patients to the right app may increase engagement by ensuring the app they are using is best suited to their mental health needs.

**Objective:**

This study aims to pilot how digital phenotyping, using data from smartphone sensors to infer symptom, behavioral, and functional outcomes, could be used to match people to mental health apps and potentially increase engagement

**Methods:**

After 1 week of collecting digital phenotyping data with the mindLAMP app (Beth Israel Deaconess Medical Center), participants were randomly assigned to the digital phenotyping arm, receiving feedback and recommendations based on those data to select 1 of 4 predetermined mental health apps (related to mood, anxiety, sleep, and fitness), or the control arm, selecting the same apps but without any feedback or recommendations. All participants used their selected app for 4 weeks with numerous metrics of engagement recorded, including objective screentime measures, self-reported engagement measures, and Digital Working Alliance Inventory scores.

**Results:**

A total of 82 participants enrolled in the study; 17 (21%) dropped out of the digital phenotyping arm and 18 (22%) dropped out from the control arm. Across both groups, few participants chose or were recommended the insomnia or fitness app. The majority (39/47, 83%) used a depression or anxiety app. Engagement as measured by objective screen time and Digital Working Alliance Inventory scores were higher in the digital phenotyping arm. There was no correlation between self-reported and objective metrics of app use. Qualitative results highlighted the importance of habit formation in sustained app use.

**Conclusions:**

The results suggest that digital phenotyping app recommendation is feasible and may increase engagement. This approach is generalizable to other apps beyond the 4 apps selected for use in this pilot, and practical for real-world use given that the study was conducted without any compensation or external incentives that may have biased results. Advances in digital phenotyping will likely make this method of app recommendation more personalized and thus of even greater interest.

## Introduction

While COVID-19 accelerated interest in mental health smartphone apps, limited patient use and engagement with these apps has emerged as a primary barrier to successful uptake [1,2]. While the challenge of limited engagement has already been well documented and ascribed to numerous causes ranging from individual patient preferences to health care system barriers, there have been fewer efforts seeking to actually improve engagement [3,4]. This study pilots 1 approach, digital navigator–guided app recommendation, to increase engagement and seeks to address methodological challenges with prior studies through objectively assessing app usage.

The challenges of low engagement with mental health apps have been well-known for nearly a decade. A landmark 2019 study [5] of 93 mental health–related smartphone apps found that the median 15-day retention rate was 3.9%. Numerous other studies confirm exponential decay in app engagement, regardless of the health condition, age, gender, or race of users [6,7]. These low engagement numbers are further exacerbated by the low initial use of mental health apps. A 2023 survey of US veterans noted that while up to 76% reported apps are important for their mental health, only 5% ever reported having tried an app at least once [8].

Yet, appreciating the challenge of app engagement does not in itself offer actionable solutions. Recent reviews have covered broad reasons why people often do not download mental health apps as well as why they rapidly stop using them if they do download them [3]. Common themes raised to boost engagement often include the need for personalized app experiences, social and therapeutic support, customization, in-app guidance, and the use of sensors to offer users real-time feedback [3]. Yet, awareness of such themes raises the question: Will implementing these themes actually increase engagement? And if such a solution can work, will that method of increasing engagement be generalizable given that over 10,000 mental health apps exist today and research specific to each unique app is not practical or feasible [9].

One promising approach toward increasing engagement is the impact of digital navigator–guided app recommendation. A digital navigator is a member of the care team trained to perform digital health roles related to equity, digital literacy, app selection, and engagement [10]. There is strong research data to suggest that patients would like guidance from clinical teams around selecting an app [11-13]. Yet, clinical teams are not aware of where to find evidence-based mental health apps and even fewer how to evaluate them [14]. Indeed, in numerous surveys, clinical teams note that they actually want education about app evaluation [13,15,16]. While several large health care systems have begun to offer app toolkits for their clinical teams to use with patients [17,18], efforts to help clinical teams recommend apps remain limited. One prior study found little impact of guidance on sustained engagement, but in this study, participants were limited to picking exercises within a single app platform [19] that subsequently was shown to suffer from low uptake or engagement [20]. A prior study by our own team found that guided app recommendation did increase engagement with apps [21] as compared to national rates.

However, no prior study has examined the impact of digital phenotyping on app recommendation. This involves accessing sensors on a patient’s smartphone to capture data related to behaviors (eg, sleep and mobility), cognition (eg, memory), and self-reported symptoms to better understand a patient’s state and use that information to match them to the best app for that state. For example, a patient who reports depression while at home may benefit from a cognitive behavioral therapy–focused app, and another who reports anxiety at work may benefit from a different app offering brief mindfulness exercises. Digital phenotyping methods can also be used to predict changes in anxiety and depression [22], meaning that it may be possible to suggest mental health app use early and as a preventive approach.

In piloting how digital phenotyping may help improve app recommendation, there are many metrics to consider. The most important may be engagement, as without engagement, even the most effective app will not be impactful. Unfortunately, recent reviews confirm that there is no standard approach to measuring engagement, with the most common method to measure the percentage of patients who complete available modules [13]. This is problematic as not all apps have modules and the completion of modules may not always signify clinically meaningful engagement. Alternative means to measure engagement include time spent in the app and subjective reports of engagement. Yet, other means to assess engagement include newer metrics like the Digital Working Alliance Inventory (D-WAI), which assesses the degree of alliance a user has to an app and has previously been shown to predict app engagement and outcomes [23]. Thus, in this study, we focused on multiple means to measure engagement with the secondary aim of assessing how the measurement of engagement itself, via subjective and objective metrics, may impact clinical outcomes.

This study seeks to improve mental health app engagement through piloting digital phenotyping–based recommendations. We hypothesize that this recommendation approach will lead to greater app engagement as compared to a control condition of participant self-selection of apps. As a secondary outcome, we explore different metrics of engagement and how different measures of engagement may inform different clinical outcomes related to app use. We hypothesize that subjective measures like self-reported engagement and D-WAI will better correlate to clinical outcomes as compared to objective measures of app use measured from screen-time logs.

## Methods

### Study Design

In this 5-week study, the first week was observational and used to gather digital phenotyping data on all participants. After this first week of data collection, all participants were randomly assigned to receive app recommendations based on their digital phenotyping data or to select an app without any assistance or data. Over the next 4 weeks, participants used their designated app and completed pre- or postintervention questionnaires via web-based study visits.

### Participants

All participants were recruited from ResearchMatch. Inclusion criteria included being aged >18 years, being proficient in the English language, being able to sign an informed consent form through a web-based process, having a primary care physician or psychiatrist, owning an Apple or Android smartphone, and scoring higher than 5 on the General Anxiety Questionnaire-7 (GAD-7) at the initial visit.

### Materials

All participants used 2 digital health apps throughout the study. The first app that every participant used was mindLAMP, an app developed by the Division of Digital Psychiatry at Beth Israel Deaconess Medical Center (BIDMC) [24]. In this study, mindLAMP served solely as a digital phenotyping data collection tool. The second app varied between participants and served as an intervention tool. Participants downloaded 1 of 4 intervention apps: UCLA Mindful (University of California Los Angeles Health), How We Feel (HWF Project Inc)., Insomnia Coach (US Department of Veterans Affairs), or Nike Training Club (Nike, Inc).

### mindLAMP App

**Figure 1:**
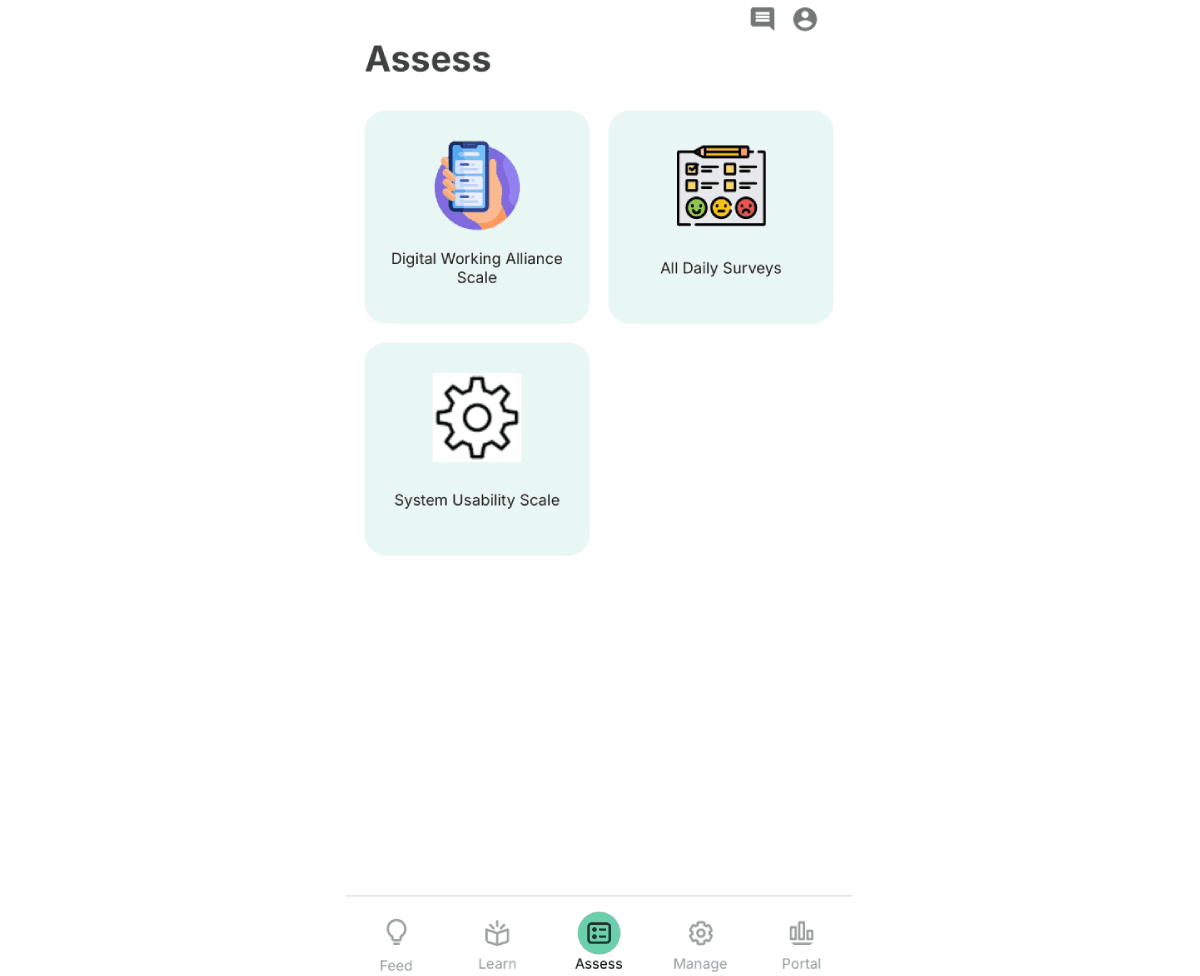
Assess tab on mindLAMP.

mindLAMP is a digital phenotyping app developed at BIDMC [24,25]. mindLAMP has a customizable interface with 5 main sections: feed, learn, assess, manage, and portal. While it can be customized to offer both interventional and data capacities, this study only used its data collection capacity including custom surveys and sensors (GPS, accelerometer, and screen use metrics; Figure 1).

### Interventional Mental Health Apps

**Figure 2:**
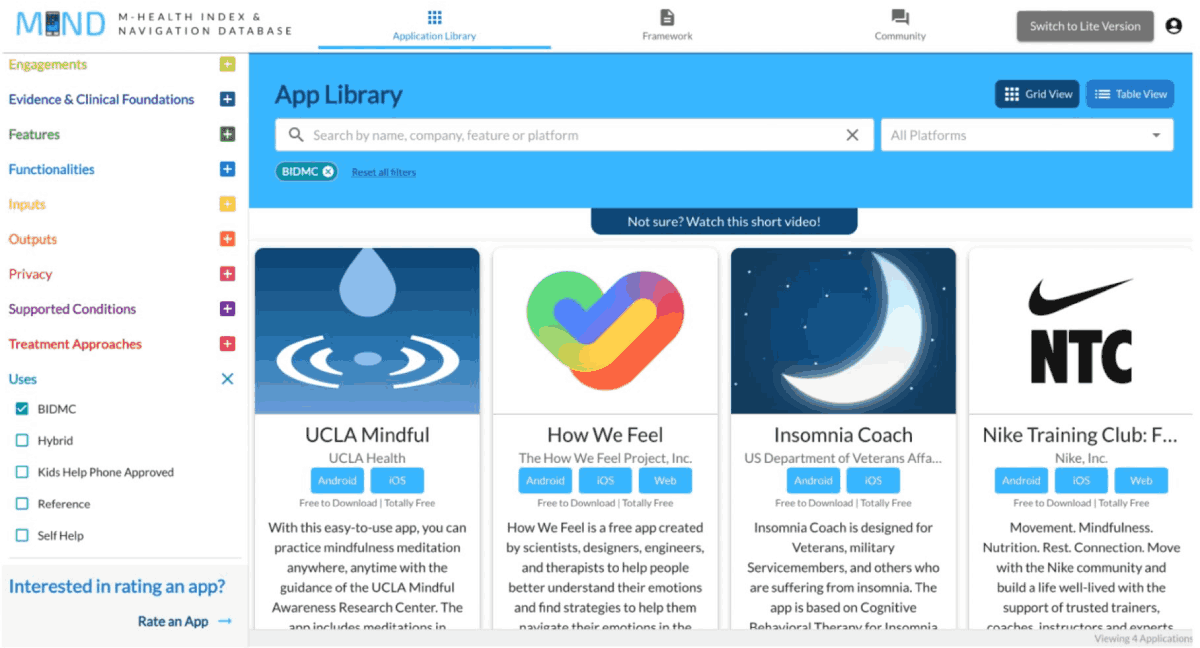
Mental health apps from MINDapps with the Beth Israel Deaconess Medical Center study filter that identified apps selected for this study. MINDapps: Mobile Health Index and Navigation Database.

All participants were designated to engage with 1 of 4 health apps for 4 weeks: UCLA Mindful, How We Feel, Insomnia Coach, and Nike Training Club. UCLA Mindful offers guided meditations for users [26]. How We Feel is a mood-tracking app that offers a range of emotions for users to choose from while also tracking aspects of their physical health such as sleep and exercise [27]. Insomnia Coach guides users with their sleep through cognitive behavioral therapy and offers weekly training with a sleep coach, tips, a log, and a diary [28]. Nike Training Club Fitness offers home workouts to healthy recipes [29]. All apps were found through the Mobile Health Index and Navigation Database (MINDapps) developed by the Division of Digital Psychiatry [30]. For this study, we created a new filter on MINDapps to display the app(s) when participants were recommended or selected an app (Figure 2). The selection of apps to include was based on feedback from patients in our clinic, advisory board, volunteer MINDapps app raters, and our prior research on app engagement [21].

### Data Collection

#### Overview

**Figure 3:**
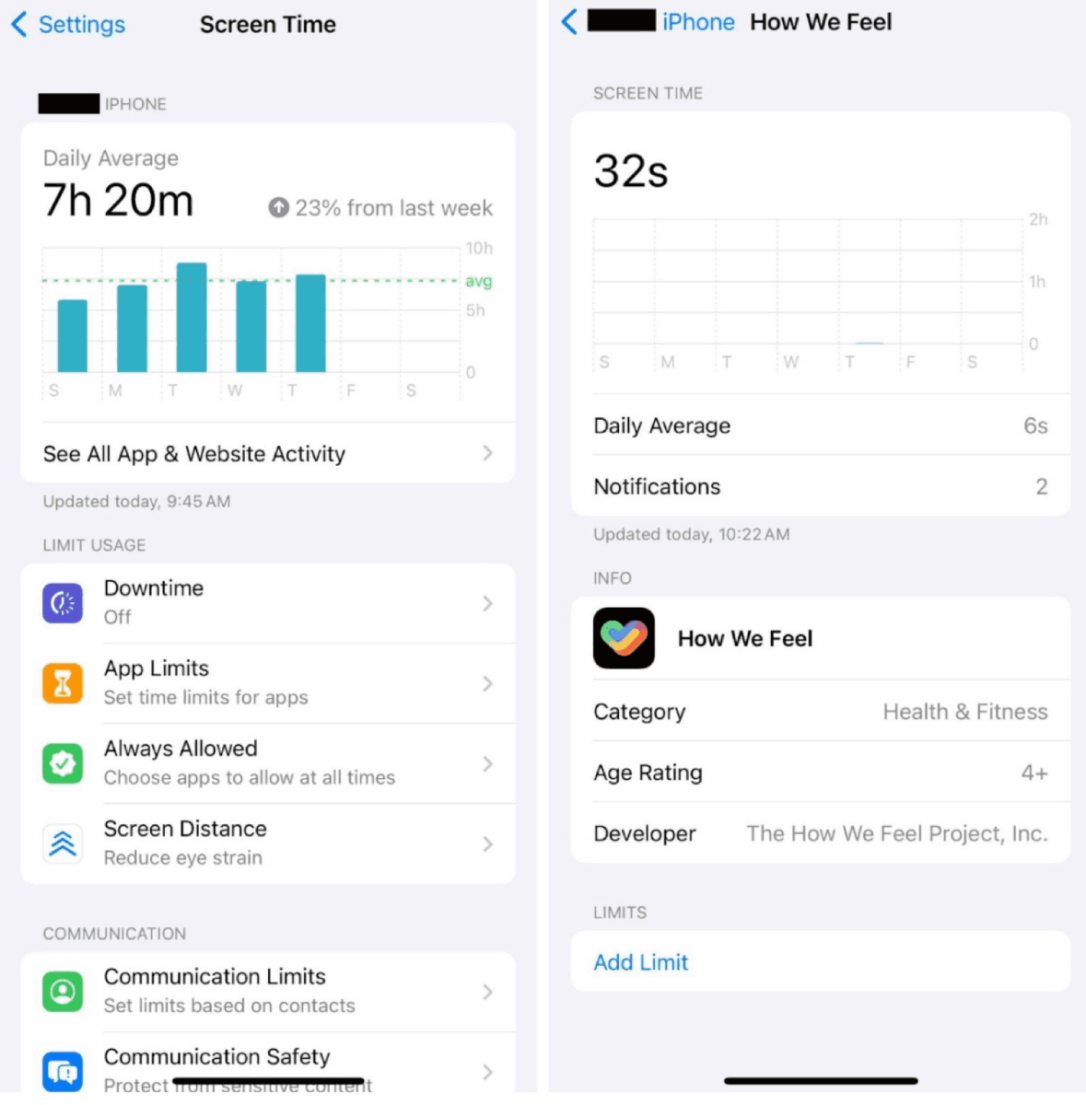
Screen Time page in the Settings app: (A) overall screen time and (B) app-specific screen time (How We Feel).

Both active and passive data features were collected as a part of this study. Passive data collection was continuously collected through the mindLAMP app for the duration of the study. Passive data specifically refers to GPS, accelerometer, phone use (eg, screen on/off and phone on/off), and step count data. Active data were categorized as survey responses and were collected at different time points. We also collected objective data on the use of each app, which were reported via the participant taking a screenshot of their screen time page in their Settings app and not possible to automatically capture with digital phenotyping across Apple and Android devices (Figure 3). Participants were asked to take screenshots of their Screen Time page at the final study visit as part of the digital data collection.

#### Active Data (Surveys)

**Figure 4:**
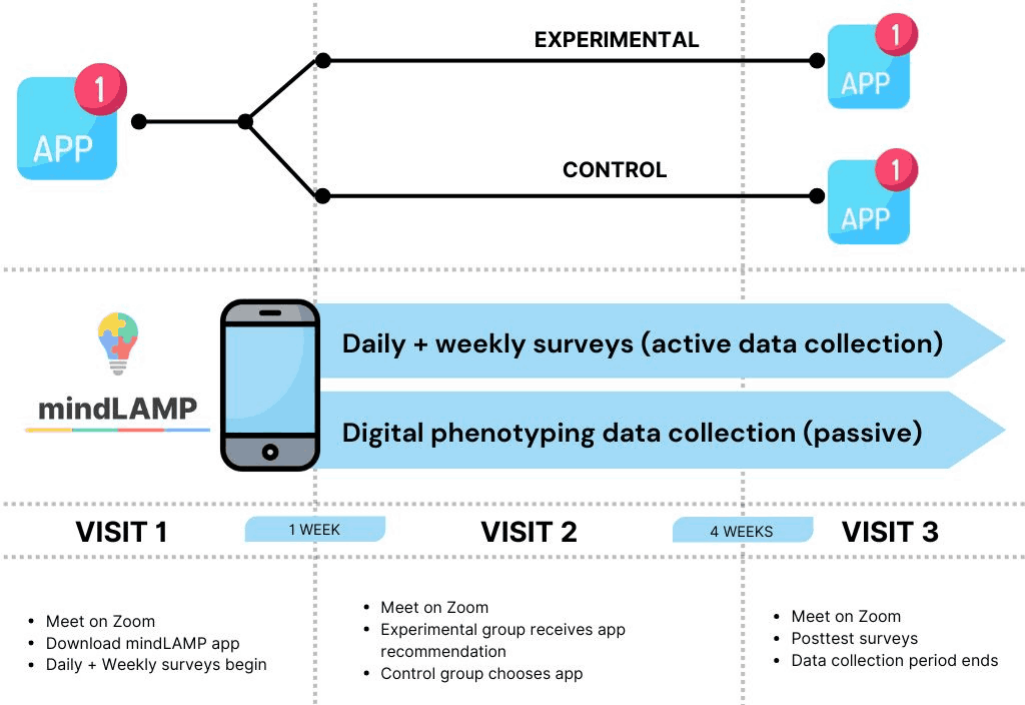
Study flow and data collection.

This study included a total of 11 surveys completed at different time points through REDCap (Research Electronic Data Capture; Vanderbilt University) and mindLAMP (Figure 4). On study visit days (3 times), participants completed a battery of standardized neuropsychiatric tests on symptoms and cognition to establish baseline, interim, and evaluation scores. The psychiatric scales consisted of GAD-7, the Insomnia Index Scale (ISI) + question about the time of sleep onset or offset, the Social Interaction Anxiety Scale (SIAS), the UCLA (University of California, Los Angeles) Loneliness Scale, the Flourishing Scale, and the Perceived Stress Scale-10 (PSS-10). During the intake appointment, researchers completed the Clinical Global Impression Scale to evaluate the participant’s illness severity. Four additional surveys were administered throughout the study: the Daily Survey, the System Usability Scale, D-WAI, and the final engagement survey. The daily survey was developed by the Digital Psychiatry research team and consisted of 6 questions to briefly assess daily activity mental health status (see Multimedia Appendix 1 for the full survey). Participants took the daily survey twice per day between visits 1 and 2. Between visits 2 and 3, participants reduced daily survey completion to 3 times per week. The System Usability Scale and D-WAI were completed during visits 1, 2, and 3. They are standardized scales used to assess app usability or perceived satisfaction and the therapeutic alliance in smartphone-based interventions, respectively [23,31]. The final engagement survey was also developed by the Digital Psychiatry team (see Multimedia Appendix 1 for the full survey) and completed on the final day of the study to understand participants’ perception of their engagement with the interventional mental health app downloaded during visit 2.

#### Group 1: Precision App Recommendation (Experimental)

**Figure 5:**
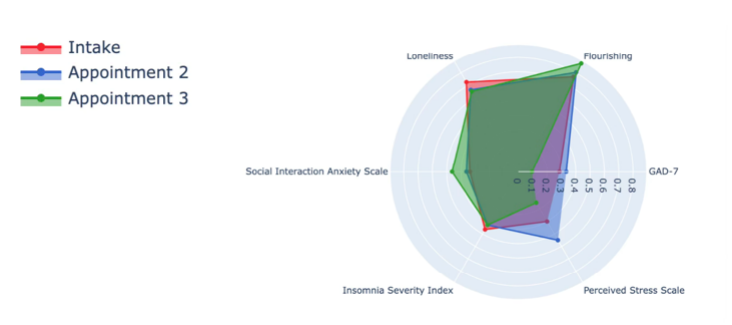
Radar plot of participant’s score on GAD-7, ISI + question about time of sleep onset/offset, Social Interaction Anxiety Scale, UCLA Loneliness Scale, the Flourishing Scale, and PSS. GAD-7: General Anxiety Questionnaire 7; ISI: Insomnia Severity Index; PSS: Perceived Stress Scale; UCLA: University of California, Los Angeles.

**Figure 6:**
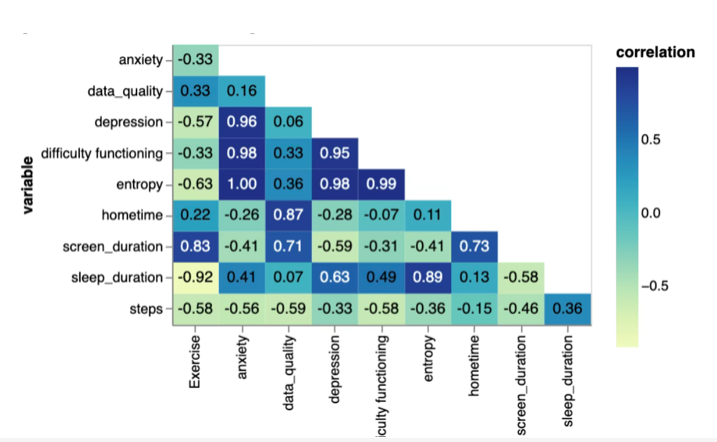
Correlation matrix displaying factors from participants’ active and passive data collected via mindLAMP. Daily questionnaires related to anxiety, depression, sleep, exercise, and difficulty functioning are correlated with passive factors such as screen duration, entropy, and hometime.

After a week of capturing digital phenotyping data about the participant, the digital navigator reviewed visualizations of those data and shared them back with the participant. The data visualizations are shown in Figures 5 and 6, consisting of a radar plot (Figure 5) and correlation matrix (Figure 6) of their data from the previous week. To recommend an app, the digital navigator assessed how mental health correlated with functioning. They selected mental health targets that featured elevated correlations for impaired functioning and persistence of this relationship over the week and ultimately recommended an app that targeted the identified symptoms.

#### Group 2: Unguided App Selection (Control)

Participants in the control group also collected digital phenotyping for 1 week, but these data were not shared back with them until after the study. They were asked for 1 week to select 1 of the same 4 mental health apps (UCLA Mindful, How We Feel, Nike Training Club, or Insomnia Coach) without guidance from their digital phenotyping data or the digital navigator. A standardized description of each app was given to the participants in the control group prior to their choosing.

#### Interventional App Use (Both Groups)

After downloading 1 of the 4 mental health apps, both groups were asked to use that app for the remaining 4 weeks of the study. With the goal of capturing naturalistic engagement, participants were not given specific instructions regarding how or when to interact with the app. Researchers instructed participants to “Engage with the app in your daily life as you see fit.” After 4 weeks, participants had their third and final meeting where their objective (screen time) and self-report (final engagement survey) engagement data were collected.

### Data Analysis Techniques

To assess if a personalized app recommendation would increase engagement effectively, we assessed correlations between self-reported engagement and objective engagement. To measure this relationship, an ordinary least squares (OLS) linear regression model was implemented using the *statsmodels* library in Python (Python Software Foundation). To assess the relationship between participants’ engagement with their respective apps and the participant’s anxiety symptoms, a series of OLS linear regressions were performed using the *statsmodels* library.

A regression model was created for every measure of engagement, which was either objective (mean app screen time) or subjective (participant’s self-reported engagement gathered from a survey). From these measures of engagement, we compared them to the change in structured surveys they took during the study. These surveys include the GAD-7, Flourishing Scale, UCLA Loneliness Scale, SIAS, ISI, and PSS-10. The specific change in structured survey scores was calculated from appointment 2 (when app use occurred) to appointment 3 (the final appointment).

### Qualitative Analysis

Following the Braun and Clarke [32] framework, a group of 5 research assistants initially reviewed the raw responses to the open-ended questions in the final engagement survey. They identified themes associated with the use and engagement of the app and added them to a table: notifications, memory, ease of use, and the content of the app. Each individual’s final engagement survey was printed out and rated by at least 2 research assistants to ensure interrater reliability. They marked where they saw the theme and indicated whether it seemed positive or negative (ie, “The notifications were annoying” → notifications → negative). In the case of dispute, an additional research assistant contributed a rating until a consensus was identified. A spreadsheet was developed to indicate themes and positive, negative, or both associations.

### Ethical Considerations

This study was approved by the BIDMC institutional review board as protocol 2022P001143. Written informed consent regarding primary and potential secondary data analyses of research data was collected and documented for all participants via the REDCap (version 14.0.30; REDCap Consortium). The protected health information of participants was securely stored on REDCap, which is a Health Insurance Portability and Accountability Act–compliant, web-based app specifically designed for research data collection and management. Study data were subsequently deidentified during the analysis process. No identification of individual participants or users is included in this paper. Participants did not receive any form of compensation for this study.

## Results

### Demographics and Groups

A total of 82 adults were recruited and enrolled in the study. There were no significant differences in sex for the control and experimental groups (*P*=1.0). There were no significant differences in baseline anxiety or depressive symptoms in each group (*P>*.05).

A total of 35 (43%) participants dropped out; 22 (27%) dropped out of the study after the first meeting, divided evenly between the treatment (n=11, 13%) and control groups (n=11, 13%). Of those participants, 9 (11%) left for unknown reasons, 6 (7%) lost interest, 3 (4%) left due to the time commitment, 3 (4%) for data quality reasons, and 1 (1%) participant left for a family emergency. After the second meeting, 13 (16%) participants dropped out (control: n=7, 8%; treatment: n=6, 7%). Two (2%) left for data quality reasons while the rest (n=11, 13%) left for unknown reasons. Table 1 below shows the full breakdown of the demographics for all 47 remaining participants.

Of note, only How We Feel and UCLA Mindful had enough participants to complete the study while using the app to produce meaningful results.

**Table 1:** Demographics of participants (n=47).

Sample characteristics			Values
**Age (years), mean (SD)**			43.0 (16)
**Sex, n (%)**			
	Female	37 (79)	
	Male	7 (15)	
	Other	3 (6)	
**Race**			
	Black or African American	2 (4.2)	
	White	42 (89)	
	Multiracial or other	3 (6)	
**Education**			
	High school graduate or GED^a^	1 (2)	
	Some college	10 (21)	
	4-year college graduate	20 (43)	
	Master’s degree or higher	14 (30)	
	Missing	2 (4)	

^a^GED: General Educational Development.

### Engagement

**Figure 7:**
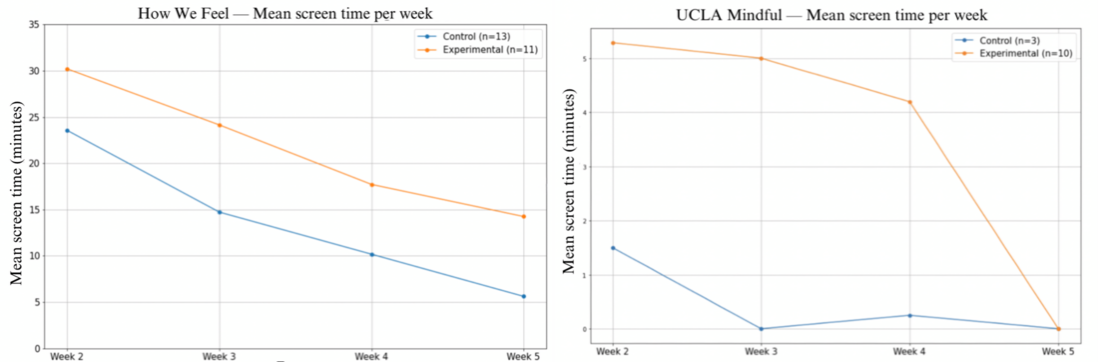
Mean screen time (in minutes) for (A) How We Feel and (B) UCLA Mindful apps in control versus experimental groups across weeks.

To determine if a personalized app recommendation would increase engagement at a population level, we plotted the mean objective engagement (mean screen time) of the control and experimental groups broken down by app (Figure 7).

#### How We Feel App

In both the control and experimental groups for the How We Feel app, mean screen time was the highest during the first week of use and steadily declined throughout the 4 weeks (Figure 7A). While statistically insignificant, the experimental group for How We Feel showed higher screen time overall (*P*=.36).

#### UCLA Mindful App

In the UCLA Mindful app group, participants in the control group barely used the app after week 2 of the study, while the experimental group tended to use the app more in the beginning, with a steep drop at the end of the study (Figure 7B).

### Self-Reported Engagement Versus Objective Engagement

The mean screen time values across apps could not be directly compared to self-reported engagement. In order to compare all apps against each other, we used the *MinMaxScaler* function from the *sklearn* Python library to map all mean screen time values to a 0-1 range for each app separately before combining the data for analysis.

Through OLS regression we found no significant correlation (*R*^2^=0.0188; *P*=.39) between self-reported engagement and scaled screen time in our pilot results. When participants were asked to rate their engagement on a scale of 1-10 (10=highest engagement), the control group’s mean rating was 6.42 (SD 2.52) as compared to the experimental group’s mean rating of 6.30 (SD 2.1).

### Engagement Versus D-WAI

**Figure 8:**
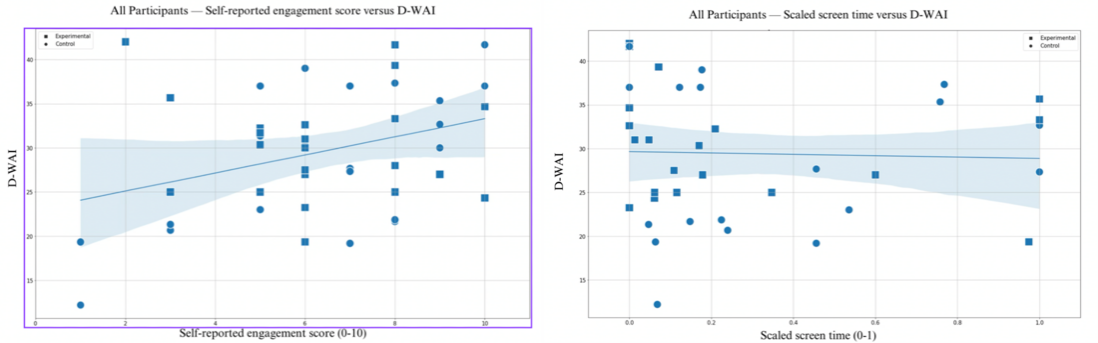
Self-reported engagement (1-10) and scaled screen time (0-1) versus mean D-WAI (0-50) for all participants and apps with regression line and 95% CI. D-WAI: Digital Working Alliance Inventory.

In addition to comparing self-reported engagement to mean screen time, we also compared both self-reported engagement and mean screen time to the participant’s mean score on the D-WAI scale (Figure 8) using the *MinMaxScaler* function noted earlier.

There was a significant positive correlation between self-reported engagement and D-WAI scores (*R*^2^=0.1199; *P*=.02; coefficient=1.0238) but no equivalent correlation between scaled screen time and D-WAI values (*R*^2^=0.0013; *P*=.83; coefficient= –0.7849). In both cases, these findings were driven more by the control group than the experimental group.

### Engagement Versus Change in Structured Survey Scores

In addition to comparing different types of engagement, a preliminary analysis compared engagement scores to changes in various clinical surveys. We explored how engagement metrics correlated with clinical symptom score changes after 1 month of app use in all participants. Overall, correlations between app engagement (via any metric) and clinical changes (via any survey) were small and most results were not statistically significant (*P>*.01). The small sample size precludes us from making any significant claims about the findings. A full table of results from our regression analyses for the How We Feel and UCLA Mindful apps can be found in Multimedia Appendix 2.

### Qualitative Results

#### Overview

**Figure 9:**
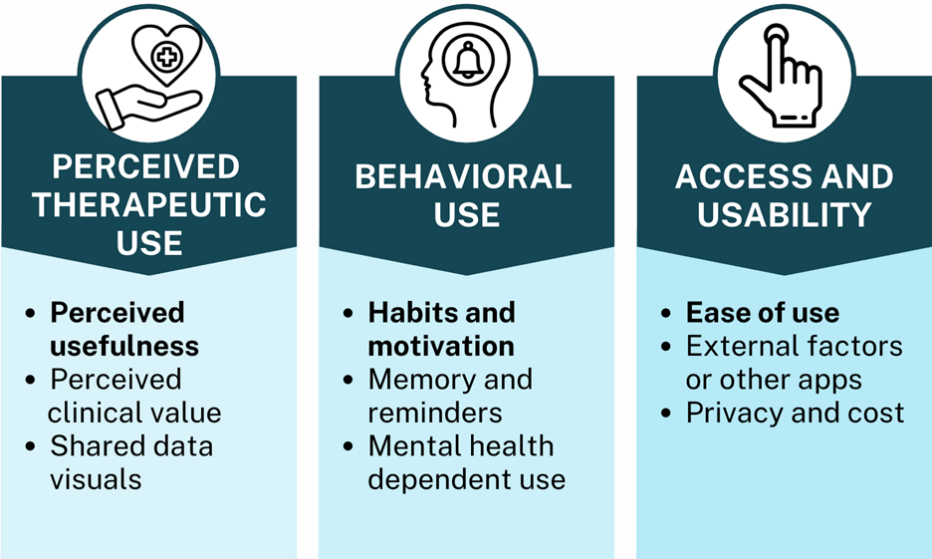
Qualitative themes.

Following the Braun and Clarke [32] framework, a team of 5 blinded coders reviewed final engagement surveys for thematic analysis to understand how users interacted with the app and what factors affected their engagement. Through qualitative analysis, the team of coders initially identified 9 independent subjects linked to engagement with the interventional mental health apps. The subjects were perceived usefulness, perceived clinical value, shared data visuals, ease of use, cost or privacy, external factors or other apps, memory or reminders, habits or motivation, and use as needed or mental health status. Next, the team further categorized the 9 topics into 3 overarching themes, including perceived therapeutic use, access and usability, and behavioral use (Figure 9). Our main themes align with a longstanding theoretical framework known as the technology acceptance model (TAM). Initially developed by Fred Davis in the 1980s, the TAM identifies what key factors influence user acceptance and subsequent engagement with a technological system [33]. The model similarly suggests the overarching role of perceived usefulness, perceived ease of use, and actual use behavior as highly relevant in determining engagement [33].

#### Perceived Therapeutic Use

Referenced 51 times in total, perceived therapeutic use, or “the degree to which an individual believes that using a particular system (app)...,” is valuable to them emerged as the most prevalent theme [33]. Participants frequently commented on the subjective usefulness of the app, the clinical benefit they perceived it to have, or the personal value of reviewing their data. These topics appeared to have positive (n=29, 57%), negative (n=15, 29%), and neutral (n=7, 12%) influences on engagement. In some circumstances, perceived usefulness drove engagement with multiple people reporting that participants found the mental health app “useful in moments of stress.” However, in some cases, it also had the opposite effect if perceived usefulness was low: “I’d have engaged more if it had more information I needed.”

#### Behavioral Use

Behavioral use, or the behavioral tendencies associated with use, was the second leading theme. Cited 44 times throughout 47 self-reports, this theme highlighted the role of memory, habit formation, and individual motivation in sustaining app use. These topics had positive (n=21, 48%) and negative (n=22, 50%) influences on engagement, with minimal neutral associations (n=1, 2%). Without motivation and a habit of use, engagement was not guaranteed in the long-term: “It seemed to have useful tools…however, I just was not self-driven enough,” and “Limited usage...mainly due to issues establishing a habit of entering data.” Participants also had conflicting opinions on the role of reminders to use the app, with some suggesting a role in increasing engagement: “I forgot to use...If I had reminders, it would have been more useful,” and others suggested against them: “App push notifications were a bit disruptive and too frequent.”

#### Access and Usability

Access and usability, encompassing the user experience and ease of use was the third most prevalent theme. Referenced 33 times in total (see Table 2), the participants commented on the fundamental factors associated with access and use of the mental health app in a positive (n=25, 76%), negative (n=7, 21%), and neutral (n=1, 3%) context. Referenced in more than half of the reports (25/33, 76%), ease of use was the most predominant subcategory under access and usability. Without being directly prompted to report on usability, the exact phrases “easy to use” or “easy to navigate“ were used in 12 independent reports. However, while access and usability emerged as importantly associated with use, it did not ensure engagement. One participant reported: “The app was intuitive to use and had a pleasing user experience, but I didn’t feel particularly engaged by it.” Another participant reported: “It was easy to use but meditation is not something that works well for me.”

**Table 2:** Qualitative analysis results.

Association	Theme		
	Perceived therapeutic utility (n=51), n (%)	Behavioral usage (n=44), n (%)	Access and usability (n=33), n (%)
Positive	29 (57)	21 (48)	25 (76)
Negative	15 (29)	22 (50)	7 (21)
Neutral	7 (12)	1 (2)	1 (3)

## Discussion

### Principal Findings

Using digital phenotyping to guide mental health app recommendations is feasible and results in higher levels of engagement over 4 weeks, although assessing the clinical impact of that higher engagement remains complex due to challenges in assessing meaningful engagement. Through qualitative analysis, we were able to better understand that the participants’ perception of engagement was driven less by perceived ease of use and perceived use and, instead, more by habit formation. These results have implications about the potential of clinical app recommendation to drive engagement, the importance of collecting both subjective and objective engagement metrics, and the role of habit formation for sustained app use.

In this study, objective engagement was highest in both groups during the first week of interventional app use and continuously decreased throughout the study period, with the largest drop from week 1 to week 2. The high engagement across both groups at the beginning suggests that common factors may drive initial engagement, but the differing course of engagement (Figure 7) suggests that distinct factors affect sustained use. As noted in the results (Figure 7), mean weekly screen time decreased nearly every week, while the digital phenotyping app recommendation group sustained higher mean screen times, suggesting the potential of this approach.

Our study results also highlight differences between objective and subjective measures of engagement. Despite having lower objective engagement scores as measured by screen time, the control group self-reported their engagement slightly higher than the digital phenotyping group. This lack of consistency between self-reported and actual use is well known [34] but rarely explored as a methodological consideration in mental health app use studies. Today, there remains no single accepted definition of engagement, with many proposals and explanations for why engagement often wanes [35,36] and methods like personalization and prompts to encourage engagement [37,38].

Inconsistencies in self-reported app use raise concerns about research methods relying solely on this as a measure of engagement. However, the value of either subjective or objective metrics of engagement is hard to determine, as there were few statistically significant correlations with any clinical changes after using these apps for 4 weeks. This negative finding could be due to the fact that the use of self-help apps is often not associated with large clinical changes and our sample size was underpowered to detect any small effects.

Our results on the D-WAI scores (Figure 8) showed a correlation with subjective, but not objective, screen time, which were considered as measures of engagement. This result is notable as prior studies have shown that this alliance metric may be a predictor of successful clinical outcomes with self-guided mental health apps but have not explored how it may impact engagement [20]. If alliance is related to subjective engagement as our results suggest, this raises mechanistic questions about how apps function and the need for further research exploring the dual role of subjective and objective factors in driving outcomes.

Additionally, our qualitative results suggest facets of a more nuanced picture of engagement beyond metrics like screen time or alliance. Participants agreed that perceived ease of use and perceived use were important factors in engaging with an app, which aligns with prior research findings grounded in TAM [22]. But while these 2 core factors were necessary for initial engagement, results suggest that sustained engagement requires the addition of habit formation. The higher rates of engagement that we saw for the digital phenotyping recommendation group may have been driven by the feedback and digital phenotyping information that could have helped participants create routines and patterns around app use.

### Limitations

Only 2 apps were picked for final analysis in order to produce meaningful results, because the other 2 apps (Insomnia Coach and Nike Training Club) did not produce an adequate sample size. The lack of uptake of those 2 apps was related to our clinical algorithm consistently recommending participants to target depression and anxiety symptoms, leading to disproportionate recommendations of their respective apps. Participants in the control group most frequently chose to download the other apps as well. The predominant self-selection of the 2 apps may be due to the high prevalence of anxiety and depression symptoms among adults, coupled with participants’ awareness that those apps targeted anxiety and depression. Additionally, both apps incorporate mindfulness techniques, an increasingly popular self-guided intervention. The visual appearance may have also influenced app selection, as it has been shown that app aesthetics play a large role in consumer appeal [39]. While we picked only 4 apps for participants to select from in this study, future studies could pick a larger number of different apps given the generalizability of this approach. Future studies should also use a method to ensure a more balanced representation across all app categories.

Another limitation of the study was the group design. The experimental group included the effect of both clinical and digital phenotypic recommendation, whereas the control group was based on a third condition of participant choice. This made it difficult to distinguish between the effect of clinical and data-based recommendations. Additionally, the control group still received app treatment, making it difficult to surmise results in the absence of intervention. Future studies should consider four distinct groups: (1) clinical recommendation; (2) digital phenotypic–based recommendation; (3) participant choice; and (4) no app (control). The small sample size (n=47) and high dropout rate (35/82, 43%) may have introduced bias and limited robust generalizability of study findings. Most participants dropped out after the first meeting for unknown reasons. A lower number of participants reported loss of interest or time commitment issues. Other participants finished the study but were excluded for data quality issues, a problem ubiquitous in digital phenotyping research. The lack of compensation and the remote nature of the study were most likely contributing factors to the small sample size and high dropout rate. Paying participants to attend study visits would have likely increased engagement but would have also confounded results. Holding in-person study visits may have decreased the dropout rate but would simultaneously made recruitment less feasible and led to a less geographically diverse sample.

The study was not designed to assess clinical impact and instead engagement. Future studies, powered appropriately, can explore if high engagement (both subjective and objective) is actually associated with improvements in depression or anxiety.

### Conclusions

Digital phenotyping app recommendation is feasible and may increase rates of engagement. However, such models need to be carefully assessed before use in larger-scale studies as they may bias recommendations toward a subset of apps. Assessing the mechanism of how this approach increases engagement, whether through digital working alliance or habit formation, can help advance the use of digital phenotyping for app recommendation.

## Appendix

Multimedia Appendix 1 Scales developed by the Division of Digital Psychiatry.

Multimedia Appendix 2 Engagement versus change in the structured survey scores.

## Abbreviations

BIDMC: Beth Israel Deaconess Medical Center
D-WAI: Digital Working Alliance Inventory
GAD-7: General Anxiety Questionnaire-7
ISI: Insomnia Index Scale
MINDapps: Mobile Health Index and Navigation Database
OLS: ordinary least squares
PSS-10: Perceived Stress Scale-10
REDCap: Research Electronic Data Capture
SIAS: Social Interaction Anxiety Scale
TAM: technology acceptance model
UCLA: University of California, Los Angeles
