# Supplementary material for: Assessing Digital Phenotyping for App Recommendations and Sustained Engagement: Cohort Study
Source: JMIR Form Res. 2024 Nov 19;8:e62725. doi: 10.2196/62725 (PMC11615540; doi:10.2196/62725)
Supplement: Multimedia Appendix 1 [file formative_v8i1e62725_app1.docx]

**Daily Survey:** The daily survey consisted of 6-items assessing anxiety, mood, sleep, and physical exercise. The anxiety and mood question were rated on a numerical scale: *Overall, how would I rate my anxiety today? (0 none - 10 worst)* and *Overall, how would you rate your mood today? (1 worst - 10 best).* The sleep questions asked approximately what time participants fell asleep the night prior and what time they woke up the morning of. Participants were also asked how much physical exercise they engaged in that day with options ranging in minutes (*<15, 15-30, 30-45, 45-60, 60+).* Additionally, participants were asked to rate how strongly you agree or disagree with this statement: *I am able to manage my day-to-day life* on a 4-point Likert scale (*0- Strongly Disagree, 4- Strongly Agree).*

**Final Engagement Survey:** The final engagement survey consisted of 4 questions, one of which was a numerical rating scale. Participants were asked to rate their engagement on a scale 1-10 (10 being the most engaging). The following open-ended question asked participants to elaborate on their experience with the app, engagement (frequency of use/nature of usage), and usefulness of the app.
