# Supplementary material for: Assessing Digital Phenotyping for App Recommendations and Sustained Engagement: Cohort Study
Source: JMIR Form Res. 2024 Nov 19;8:e62725. doi: 10.2196/62725 (PMC11615540; doi:10.2196/62725)
Supplement: Multimedia Appendix 2 [file formative_v8i1e62725_app2.docx]

**Engagement vs. Change in Structured Survey Scores for How We Feel App**

**Table 2A**: Proportion of variation in clinical score changes explained by engagement metric for participants using the How We Feel app.

| Clinical Survey Δ | Self-Reported Engagement | | Mean Screentime | | Mean D-WAI | |
| --- | --- | --- | --- | --- | --- | --- |
|  | R^2^ | p-value | R^2^ | p-value | R^2^ | p-value |
| GAD-7 | 0.0311 | 0.4101 | 0.0033 | 0.8052 | 0.0174 | 0.5691 |
| Flourishing | 0.0125 | 0.6035 | 0.0000 | 0.9825 | 0.0589 | 0.2892 |
| UCLAQ | 0.0090 | 0.6595 | 0.0272 | 0.475 | 0.0003 | 0.9384 |
| SIAS | 0.0001 | 0.9704 | 0.2171 | *0.03326* | 0.0223 | 0.5179 |
| ISI | 0.1615 | 0.05158 | 0.1753 | 0.05889 | 0.1397 | 0.09512 |
| PSS-10 | 0.0462 | 0.3132 | 0.0540 | 0.3108 | 0.1386 | *0.09656* |

**Engagement vs. Change in Structured Survey Scores for UCLA Mindfulness App**

**Table 2B:** Proportion of variation in clinical score changes explained by engagement metric for participants using the UCLA Mindful app.

| Clinical Survey Δ | Self-Reported Engagement | | Mean Screentime | | Mean D-WAI | |
| --- | --- | --- | --- | --- | --- | --- |
|  | R^2^ | p-value | R^2^ | p-value | R^2^ | p-value |
| GAD-7 | 0.0016 | 0.9015 | 0.3419 | 0.09818 | 0.1907 | 0.1558 |
| Flourishing | 0.0862 | 0.3545 | 0.0882 | 0.4378 | 0.0796 | 0.3744 |
| UCLAQ | 0.1010 | 0.314 | 0.0607 | 0.5228 | 0.1452 | 0.2217 |
| SIAS | 0.0680 | 0.413 | 0.1705 | 0.2694 | 0.0277 | 0.6051 |
| ISI | 0.4281 | *0.02098* | 0.0263 | 0.677 | 0.5906 | *0.003494* |
| PSS-10 | 0.0289 | 0.5974 | 0.0389 | 0.6112 | 0.1247 | 0.2603 |
